# Supplementary material for: Effects of a Novel Infant Formula on the Fecal Microbiota in the First Six Months of Life: The INNOVA 2020 Study
Source: Int J Mol Sci. 2023 Feb 3;24(3):3034. doi: 10.3390/ijms24033034 (PMC9917896; doi:10.3390/ijms24033034)
Supplement: Supplementary file 1 [file ijms-24-03034-s001.zip › ijms-2151279-supplementary.pdf]

Effects of a Novel Infant Formula on the Fecal Microbiota in the First Six Months of Life: the INNOVA 2020 study

Francisco Javier Ruiz-Ojeda[1,2,3,4,#], Julio Plaza-Diaz[1,2,5,#], Javier Morales[6], Guillermo Álvarez-Calatayud[7], Eric Climent[8], Ángela Silva[8], Juan F. Martinez-Blanch[8], Maria Enrique[8], Marta Tortajada[8], Daniel Ramon[8], Beatriz Alvarez[8], Empar Chenoll[8], Ángel Gil[1,2,4,9,*]

[1]Department of Biochemistry and Molecular Biology II, School of Pharmacy, University of Granada, 18071, Granada, Spain

[2]Instituto de Investigación Biosanitaria IBS.GRANADA, Complejo Hospitalario Universitario de Granada, Granada 18014, Spain

[3]RG Adipocytes and Metabolism, Institute for Diabetes and Obesity, Helmholtz Diabetes Center at Helmholtz Center Munich, Neuherberg, 85764 Munich, Germany.

[4]Institute of Nutrition and Food Technology "José Mataix", Centre of Biomedical Research, University of Granada, Avda. del Conocimiento s/n. 18016 Armilla, Granada, Spain

[5]Children's Hospital of Eastern Ontario Research Institute, Ottawa, ON K1H 8L1, Canada.

[6]Product Development Department, Alter Farmacia SA, 28880 Madrid, Spain.

[7]Gregorio Marañon Maternal and Children's Hospital.

[8]ADM-BIOPOLIS. 46980 Paterna, Valencia, Spain

[9]CIBEROBN (CIBER Physiopathology of Obesity and Nutrition), Instituto de Salud Carlos III, 28029 Madrid, Spain

*Correspondence: agil@ugr.es; Tel.: +34 695466922; (A.G.)

[#]These authors contributed equally to this work

Supplementary table S1. Nutritional composition of the standard infant formula (STD) and study formula (INN).

Composition	STD formula			INN formula		
	100 g	100 mL	100 kcal	100 g	100 mL	100 kcal
Energy	514	67		489	67	
Energy	2152	280		2046	279	
Total Fat	27	3.5	5.2	25.6	3.5	5.2
Linoleic acid (ω-6)	3.79	493	736	3.147	427	641
α-linolenic acid (ω-3)	500	65	97	387	53	79
Araquidonic acid AA (ω-6)	53	6.9	10	118	16	24
Docosahexaenoic acid DHA (ω-3)	53	6.9	10	118	16	24
Carbohydrates	56.4	7.3	11	54.6	7.4	11.1
Total sugars	56.4	7.3	11	53	7.2	10.8
Lactose	55	7.2	10.7	53	7.2	10.8
Fiber	1.3	0.18	0.22	1.7	0.22	0.33
Proteins	10.6	1.4	2.1	9.4	1.3	1.9
Whey proteins	6.4	0.83	1.2	6.6	0.9	1.3
Caseins	4.2	0.55	0.82	2.8	0.38	0.57
α-casein	20 % of total protein			8% of total protein		
β-casein	14 % of total protein			18 % of total protein		

Supplementary table S2. Relative abundances at the species levels of fecal bacteria in infants fed the INNOVA (INN) or a standard formula (STD) compared to exclusive breastfed (BF) infants up to 6 months of age

Species	2 months			6 months		
	BF (n=64)	STD (n=64)	INN (n=63)	BF (n=52)	STD (n=55)	INN (n=52)
Bifidobacterium breve	3.2 (0 - 95.2)[a]	0.3 (0 – 7.0)[b]	1.1 (0 – 59.0)[a]	1.8 (0 - 38.3)	0.7 (0 - 20.3)	1.2 (0 - 52.3)
Bifidobacterium longum	47.4 (0.7 - 99.7)[a]	55 (1.4 - 99.3)[b]	79.3 (3.5 - 99.8)[b]	58.2 (2.0 - 99.5)	63.2 (0.4 - 98.1)	77.0 (24.9 - 99.2)
Bifidobacterium bifidum	19.7 (0.04 - 95.7)	7.0 (0.03 - 66.7)	6.9 (0.03 - 95.7)	22.6 (0.05 - 92.1)[a]	8.9 (0.1 - 52.6)[b]	9.4 (0.03 - 47.9)[b]
Akkermansia muciniphila	0.3 (0 - 2.8)	5.1 (0 - 92.1)	1.4 (0 - 74.3)	1.0 (0 - 30.3)[a]	3.0 (0 - 86.5)[b]	1.6 (0 - 36.7)[a]
Ruminococcus gnavus	0.2 (0 - 90.7)	0.2 (0 – 73.8)	0.08 (0 -10.1)	0.2 (0.01 - 40.9)[a]	1.6 (0 – 46.7)[b]	0.2 (0 – 73.9)[a]
Clostridium difficile	0.04 (0 - 46.7)[a]	0.07 (0 – 24.9)[b]	0.03 (0 - 8.4)[a]	0.04 (0 - 5.7)[a]	0.3 (0 – 24.2)[b]	0.02 (0 – 39.3)[a]
Lactobacillus paracasei	0.02 (0 - 2.5)[a]	0 (0 – 0.2)[b]	0.01 (0 – 0.3)[a]	0 (0 - 0.3)	0 (0 – 0.1)	0 (0 – 0.5)
Staphylococcus aureus	0.01 (0 - 0.4)[a]	0 (0 – 0.1)[b]	0.01 (0 – 0.3)[a]	0 (0 - 0.09)	0 (0 – 0.03)	0 (0 – 0.07)
Streptococcus salivarius	0.1 (0 – 1.4)[a]	0.05 (0 – 0.6)[b]	0.1 (0 -1.2)[a]	0.09 (0 - 1.4)	0.01 (0 – 0.9)	0.05 (0 – 0.7)

Data are expressed as median and range. Labeled medians with identical letters are not significant. Different letters means significant differences ($P < 0.05$).

Supplementary figure S1. Absolute counts represented by log-fold change between visit 4 (6 months) and visit 1 (21 days). A phylum, B genus by log-fold change are visualized by divergent bar plots.

A

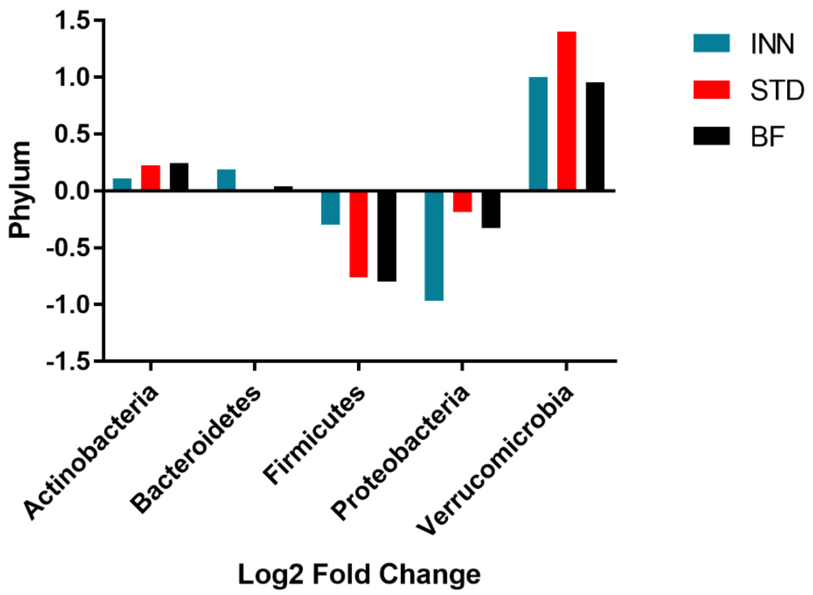

B

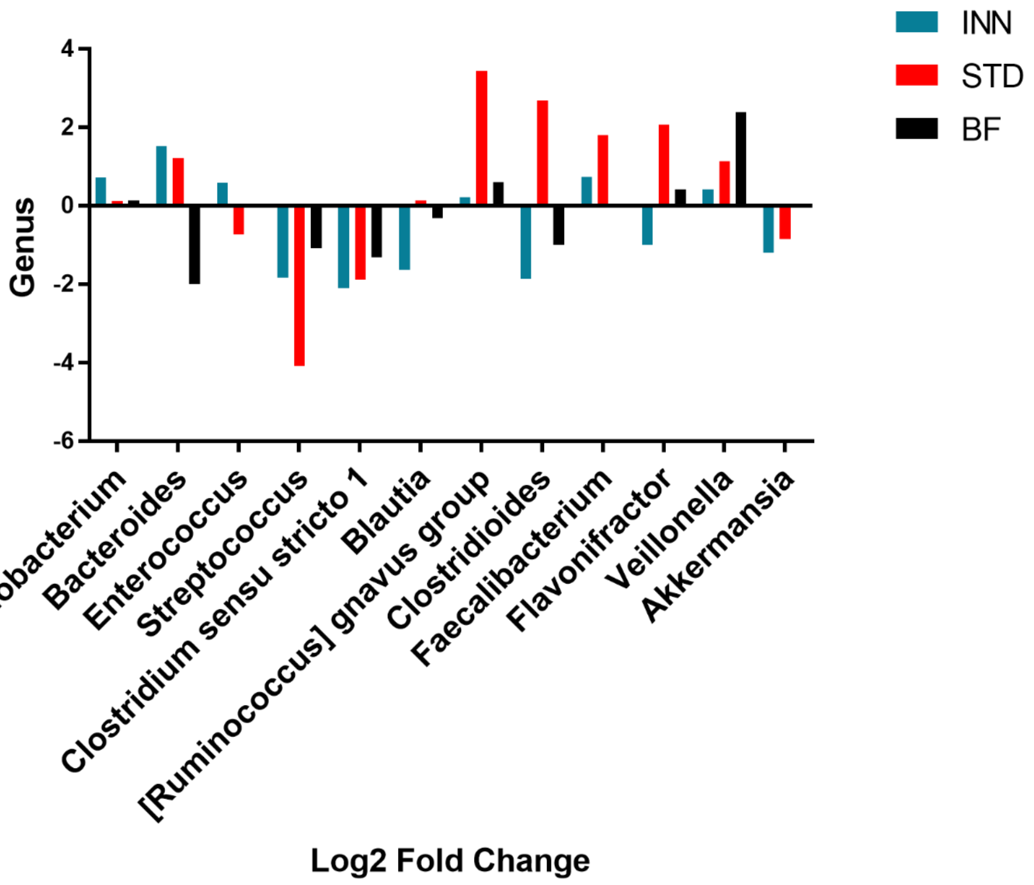
INN
STD
BF
4
2
0
-2
-4
-6
Genus
Bifidobacterium
Bacteroides
Enterococcus
Streptococcus
Clostridium sensu stricto 1
Blautia
[Ruminococcus] gnavus group
Clostridioides
Faecalibacterium
Flavonifractor
Veillonella
Akkermansia
Log2 Fold Change

Supplementary figure S2. Correlations between bacterial diversity indices, bacterial variables, SCFAs levels, metabolic traits and clinical outcomes A: Innova formula group; B Standard formula group; C: Breastfed group

A

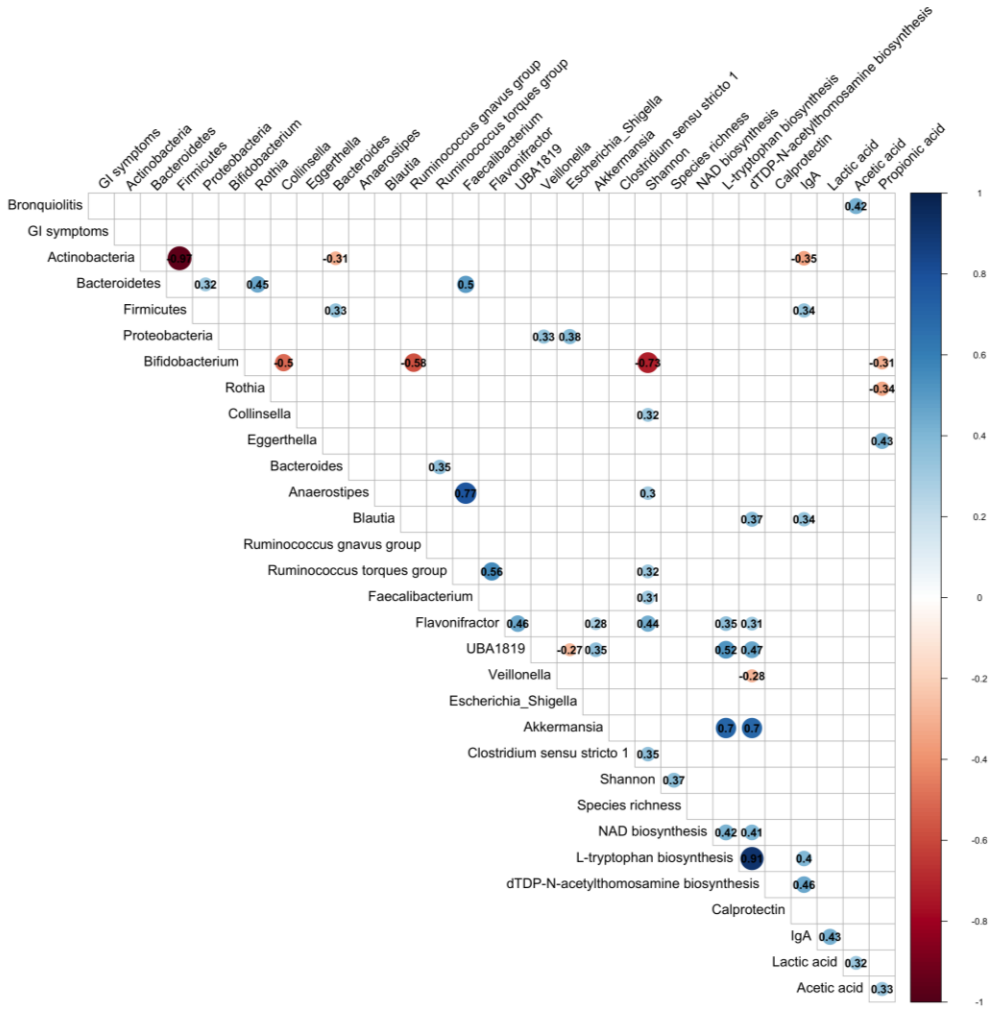

B

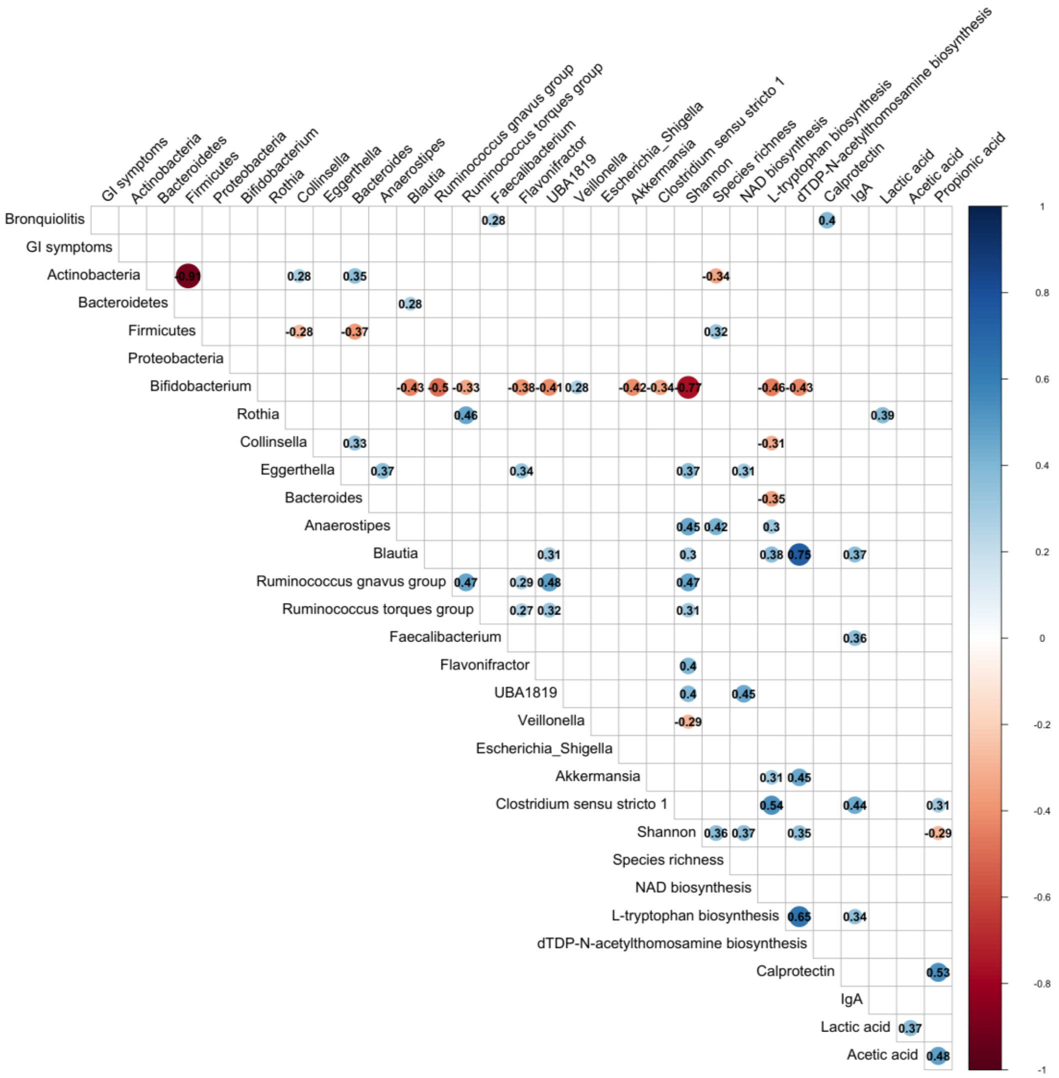

Bronquiolitis
GI symptoms
Actinobacteria
Bacteroidetes
Firmicutes
Proteobacteria
Bifidobacterium
Rothia
Collinsella
Eggerthella
Bacteroides
Anaerostipes
Blautia
Ruminococcus gnavus group
Ruminococcus torques group
Faecalibacterium
Flavonifractor
UBA1819
Veillonella
Escherichia_Shigella
Akkermansia
Clostridium sensu stricto 1
Shannon
Species richness
NAD biosynthesis
L-tryptophan biosynthesis
dTDP-N-acetylthomosamine biosynthesis
Calprotectin
IgA
Lactic acid
Acetic acid
Propionic acid

C

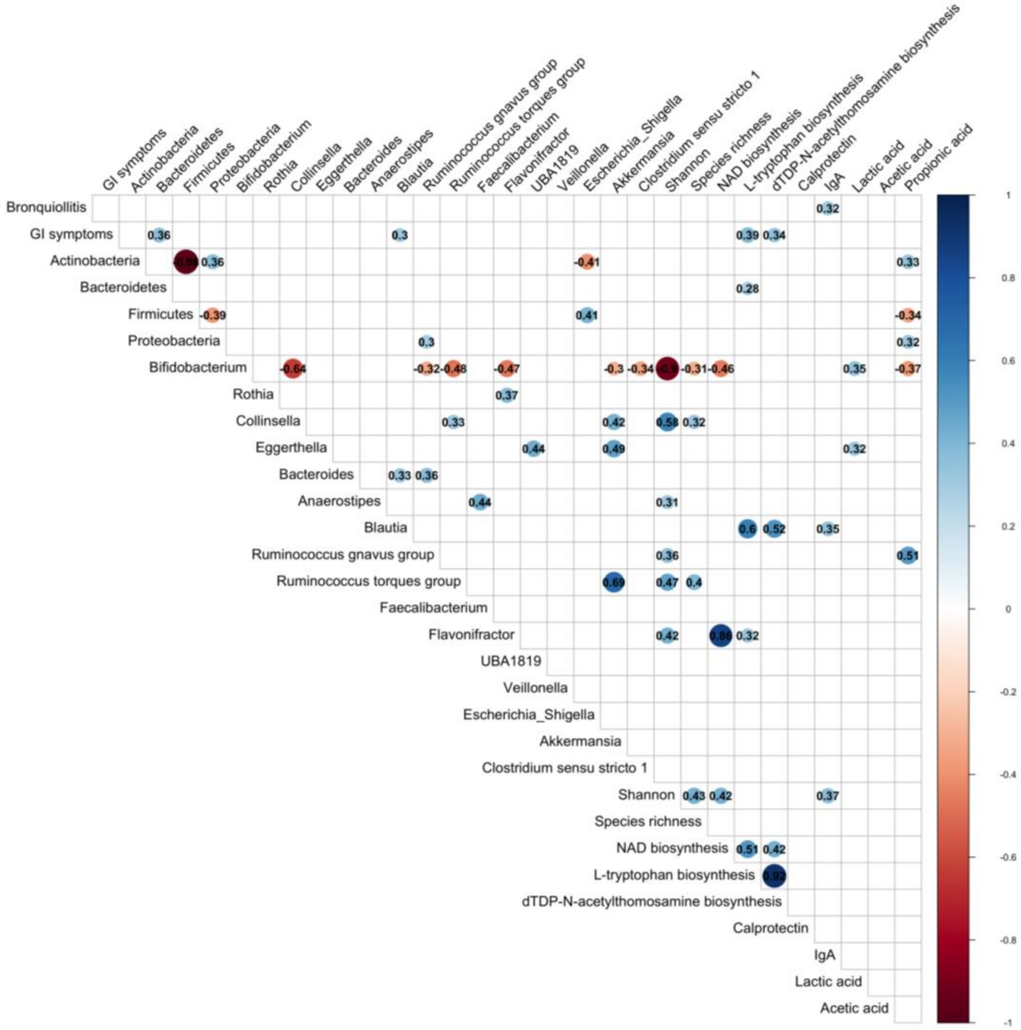
Bronquiollitis
GI symptoms
Actinobacteria
Bacteroidetes
Firmicutes
Proteobacteria
Bifidobacterium
Rothia
Collinsella
Eggerthella
Bacteroides
Anaerostipes
Blautia
Ruminococcus gnavus group
Ruminococcus torques group
Faecalibacterium
Flavonifractor
UBA1819
Veillonella
Escherichia_Shigella
Akkermansia
Clostridium sensu stricto 1
Shannon
Species richness
NAD biosynthesis
L-tryptophan biosynthesis
dTDP-N-acetylthomosamine biosynthesis
Calprotectin
IgA
Lactic acid
Acetic acid
Propionic acid
